# The effects of polyunsaturated fatty acids in alcohol dependence treatment - a double-blind, placebo-controlled pilot study

**DOI:** 10.1186/1472-6904-11-10

**Published:** 2011-07-26

**Authors:** Marina N Fogaça, Ruth F Santos-Galduróz, Jaqueline K Eserian, José Carlos F Galduróz

**Affiliations:** 1 Psychobiology Department, Federal University of São Paulo, Edifício de Ciências Biomédicas, Rua Botucatu 862 - 1° Andar, São Paulo, Brasil; 2 Laboratory of Physical Activity and Aging of Rio Claro Biosciences Institute - Universidade Paulista Júlio de Mesquita Filho, Rio Claro, São Paulo, Brasil and Centro de Matemática, Computação e Cognição da UFABC, Santo Andrá, São Paulo, Brasil; 3 Psychobiology Department, Federal University of São Paulo, Edifício de Ciências Biomédicas, Rua Botucatu 862 - 1° Andar, São Paulo, Brasil

## Abstract

**Background:**

The lipid fraction of cell membranes consists of polyunsaturated fatty acids (PUFAS), and chronic alcohol use alters it, modifying its permeability, what might contribute for the dysfunctional metabolism observed in the central nervous system of alcohol dependent patients. Therefore, the supplementation of PUFAS can be an important adjuvant in alcoholism treatment.

**Methods:**

This was a placebo controlled, double blind, randomized study where, 80 alcohol dependent patients, according to DSM-IV, were allocated in four groups with 20 patient each: 'PUFAS', 'Naltrexone', 'Naltrexone + PUFAS' and 'Placebo'. Those substances were administered for 90 days and scales were applied to assess patients craving (OCDS) and alcohol dependence severity (SADD) at baseline and after 90 days. PUFAS serum levels were assessed before and after treatment by high performance liquid chromatography assay.

**Results:**

Forty-three patients completed the trial. There was a significant improvement over time on drinking days, SADD and OCDS scores in all groups (p < 0.001). The drinking days comparison between groups did not show statistical significant difference. The same effect was observed for compulsion (OCDS) and severity of dependence scale (SADD). The serum levels of PUFAS increased in all the supplemented groups after treatment, although not significantly.

**Conclusions:**

The oral supplementation of 2 g PUFAS for 3 months did not significantly differ from placebo in reducing the amount of alcohol ingestion, or OCDS and SADD scores in a group of alcohol dependent patient.

**Trial registration:**

NCT01211769

## Background

Polyunsaturated fatty acids (PUFAS) are unsaturated fatty acids whose carbon chain has more than one double bond per molecule. Of those, omega-3 (n-3) and omega-6 (n-6) are known as "essential" fatty acids, as humans are unable to synthesize them.

The n-3 series are derived from alpha-linolenic acid (ALA) and the n-6 series, from linoleic acid (LA). The main ALA derivates are eicosapentaenoic acid (EPA) and docosahexaenoic acid (DHA). Gamma linolenic acid (GLA), dihomogamma linolenic acid (DGLA) and arachidonic acid (AA) are the main LA products. GLA is produced from LA by the enzyme delta-6-desaturase and is further metabolized to DGLA. A small amount of DGLA can also be converted to AA by the enzyme delta-5-desaturase. Human conversion of ALA and LA to their derivates is limited, and the existence of them depends on the ingestion of certain food sources.

PUFAS are key components to the brain and count up to 15-20% of its dry mass [1]. Other relevant roles of PUFAS are the influence over fluidity of neuronal membranes and acting as second messengers in neurotransmitter systems [2]. According to Naliwaiko, et al. [3], changes in cell membrane fatty acid composition occurs in the central nervous system (CNS) depending on PUFAS dietary intake [4,5]. Therefore it has been postulated that adequate supplementation of omega-3 and omega-6 would have beneficial effects on brain functions, although the ratio between them has not yet been established [6].

Omega-3 fatty acids have been associated with several benefits. This is particularly true for two essential fatty acids of the omega-3 family; docosahexaenoic acid (DHA) and eicosapentaenoic acid (EPA). As an example, it has been used as an adjuvant treatment of systemic hypertension, Crohn's disease, rheumatoid arthritis, age-related macular disease and asthma with favorable outcomes. There are also reports of effectiveness in reducing risk of primary cardiac and promising data on the prevention of breast and lung cancer [7,8].

In psychiatry, the strongest evidence of the beneficial effects of omega-3 is in mood disorders. A meta-analysis involving patients with major depressive disorder and bipolar disorder provided evidence that 1 to 2 grams daily of omega-3 PUFA supplementation can reduce symptoms of depression [9]. Furthermore, it has been suggested that supplementation with eicosapentaenoic acid may be more beneficial than that of docosahexaenoic acid in these disorders [10-12]. Although several factors do not allow definitive conclusions about what kind of omega-3 would bring better results. Other studies also support the action of PUFAS in reducing the number of relapses of cocaine addicts, minimizing the aggressiveness [13,14], improving bipolar disorders [15] and borderline personality disorder symptoms [16,17].

In 1987, Glen et al. summarized alcohol effects in PUFAS metabolism. He stated that ethanol consumption leads to an increase in the ratio of linoleic to arachidonic acid in the phospholipids of tissues. Some of the main contributors for this PUFAS altered ratio would be a reduced intake, absorption, and alteration in fatty acid metabolism [18]. Alcohol also inhibits ∂-6 and ∂-5-desaturases, enzymes associated with the conversion of linoleic acid to gamma linolenic (GLA), and dihomogamma linolenic acids to arachidonic acid [19]. Moreover alcohol has a direct effect on cell membranes composition. A relative increase in n-6 PUFAS in the membranes increases the fluidity resulting in cell damage. Interestingly, it has been reported that this pathological effect could be reduced, at least partially by fatty acid supplementation, especially n-3 PUFAS [18].

Previous studies have evaluated the role of PUFAS in alcoholism. They are linked to the action of neurotransmitters [20], liver damage produced by alcohol [21,22], their effects on tolerance [23] and even as attenuators of the negative effects of chronic alcohol use [24]. Treatment with omega-3 looks promising, but few studies have been published so far. Further studies are needed to identify individuals likely to be benefited by this type of treatment, assess the durability of these effects and to determine dosage and treatment time recommended [25].

There is no literature to date, associating PUFAS as a potential treatment to prevent compulsion for alcohol. This association seems plausible, since the lipid fraction of cell membranes consists of PUFAS, and chronic alcohol use alter the absorption of these acids, modifying its permeability. The aim of this study was to investigate the effects of PUFAS in alcohol-dependent patients, and to evaluate the possible reduction in craving for alcohol.

## Methods

The present study is a placebo controlled, double blind, randomized trial. It was approved by the Institutional Board Review (IRB) at Federal University of Sao Paulo - UNIFESP (CEP 0185/06).

### Patients Selection

The research was announced in the media (radio and Metro newspaper) and Federal University of Sao Paulo internal network, and followed by an initial selection of volunteers (initial telephone contact). For this initial screening, the inclusion criteria were: male subjects, aged between 30 and 50, dependent of alcohol according to DSM-IV [26], with no history of known clinical disease, Axis I psychiatric comorbidities, or other substance misuse in the past month (except for tobacco).

After the screening, patients were invited to a clinical interview. The interview included medical and psychiatric history and physical examination. Following the interview, volunteers underwent laboratorial workup which consisted of: uric acid, albumin, amylase, bilirubin and fractions, calcium, phosphorus, total cholesterol, HDL, LDL, creatinine, alkaline phosphatase, fasting glucose, complete blood count, lipid profile T3, T4 and TSH, SGOT, SGPT, triglycerides, urea, urinalysis, VDRL, Serologic tests for (Hepatitis A, Hepatitis B, Hepatitis C, Chagas disease and Elisa for HIV), Screening for drugs of abuse (cocaine, THC, methamphetamine, barbiturates, phencyclidine, amphetamine, morphine and benzodiazepines).

The volunteers eligible to participate in the study received written information pertinent to the research, including the safety aspects and risks of the administered substances, and signed the informed consent.

### Treatment arms

After selection patients were randomized into four groups: 'Placebo', 'Naltrexone', 'PUFAS' and 'Naltrexone + PUFAS'. The composition of PUFAS capsules was of 1 g *Borago officinalis *oil (rich in omega 6) and another of 1 g of fish oil (rich in omega 3). The capsules were provided from Herbarium Laboratório Botânico (Colombo, PR, Brazil) and contained respectively 120 mg of GLA and 400 mg EPA + DHA, which corresponds to a ratio of approximately (3, 5: 1) between omega-3 and omega-6, similar to the previous ratio suggested benefic [6]. The fish oil capsule composition was 160 mg of EPA and 240 mg of DHA. Capsules of liquid paraffin composed the placebo version of PUFAS. Cristália Produtos Químicos Farmacêuticos LTDA (Itapira, SP, Brazil) provided Naltrexone 50 mg and also identical placebo-Naltrexone pills.

The medications were administered daily, after an initial period of seven days of detoxification. Patients were then followed monthly for 90 days, allowing observation of possible side effects that might implicate in volunteers exclusion from the study. Patient returned the empty bottles of medication on each visit (end of first, second and third month), as well as a calendar with the marked days in which they took the medication.

### Follow-up measurements

The serum level of PUFAS (EPA, DHA, AA and Linoleic acid) was dosed at the baseline and last day of follow-up. The quantification protocol of PUFAS dosage was conducted as described by Naliwako et al [3] using the high performance liquid chromatography technique (HPLC). Briefly, total lipids were extracted from plasma, using chloroform-methanol (2:1 vol/vol). Fatty acids were derivatized with 4-bromomethyl-7-coumarin and then separated on a Shimadzu LC-10A high performance liquid chromatograph using an octa- decylsilica column (25 cm £ 4:6 mm i.d.; particle size 5 mm). Fatty acids were resolved isocratically using a mobile phase of acetonitrile-water (gradient from 77:23 to 90:10 vol/vol) at a flow rate of 1 ml/min. Fatty acid derivatives were detected by fluorescence (325-nm excitation; 395-nm emission).

During the study, two scales were applied to assess patients craving and alcohol dependence severity, at the baseline visit and three months after the initiation of treatment. The SADD questionnaire (Short Alcohol Dependence Data), a self-completion questionnaire designed to evaluate the presence and the degree of severity of alcohol dependence, adapted and validated for use in Brazil by Jorge & Masur [27]; and the OCDS Scale (Obsessive-Compulsive Drinking Scale), also a self rating scale, looking at obsessive thinking and compulsive desire to use alcohol.

### Statistical Analysis

Continuous variables were tested by means of tests of normality, to verify pre-requisites for the implementation of parametric tests. Shapiro Wilk tests were performed for normality and variables without normal distribution were tested with non-parametric tests. To assess whether in baseline, the groups had similar results in relation to the "drinking days" and measures of the scales SADD and OCDS, we used the non-parametric test Kruskal-Wallis, which showed no statistical difference between groups. To compare if there were differences on Drinking Days or SADD and OCDS scales between the groups and over the time of follow-up, repeated measures ANOVA were performed.

To assess if there was any difference between the analyzed groups, scores of SADD and OCDS were ranked, and chi-square tests were performed. SADD questionnaire was ranked in "low dependence" (1 - 9), "medium dependence" (10 - 19) and "high dependence" (20 - 45), as previously reported by Raistrick *et al *[28]. The OCDS scores were separated in "insignificant" (0 - 5), "low craving" (6 - 14), "medium craving" (15 - 25) and "severe craving" (26 and above), as in previous publication [29].

The statistical software Statistica for Windows (version 9.0) was used to perform the above-mentioned analysis.

## Results

A total of 80 patients, 20 per group were enrolled. The groups did not differ at the baseline from demographic characteristics, such as: marital status, educational level or employment status. At the end of three moth follow-up period, 43 patients were still in the study: PUFAS (n = 12), naltrexone (n = 11), naltrexone + PUFAS (n = 9) and Placebo (n = 11). Thirty-seven patients were excluded: 30 due to incomplete follow-up, 2 for clinical complications and 5 were referred to inpatient unit care.

All groups improved at the end of 3 months. The repeated measures ANOVA showed significant effect of time in relation to "drinking days", SADD and OCDS in all groups (p < 0.001). At the beginning of the study, patient referred to have drunk in 20.49 (± 9.78) of the 30 days previous to the scheduled appointment. In the final visit it was reduced to 7.95 days (± 10.80).

As for SADD questionnaire, the baseline score was 33.17 (± 10.39) and the final measure was 10.86 (± 13.49). Dependence severity was ranked as: severe dependents 90% of the patients (n = 72), moderate dependents 9% (n = 7) and only one patient was considered to have a low severity. Remarkably, in the final appointment, of the 43 patients that remained in the study, 23% were still severe dependents (n = 10), 14% had a moderate classification (n = 6), 28% were ranked as low dependents (n = 12) and 35% did not point at all in the questionnaire (n = 15). A Chi-square test performed did not show any difference between the groups according to the SADD analysis.

Regarding the OCDS scale, in the baseline assessment, patient had a mean score of 40.81 (± 13.07) and 14, 67 (± 16.33) in the final visit. When ranked, 72 of the 80 patients were considered to have a strong craving, 5 patient a moderate and 3 were classified as low in the baseline moment. When OCDS was last performed, the 43 patients had their craving ranked respectively as strong 21% (n = 9), moderate 16% (n = 7), low 19% (n = 8) and insignificant 64% (n = 19). Again, the Chi-square test was performed, and no difference was observed between the studied groups.

There was no statistical significant difference in the drop-out rate between the studied groups (PUFAS, Placebo, Naltrexone and Naltrexone + PUFAS). There was a statistical significant difference of treatment completion in "drinking days" (p = 0.03). The mean "drinking days" in the last month of patient that completed the trial was 18.46 (± 1.46), while in the "drop-out" group, it was of 23.08 (± 1.57). There was no statistical difference of SADD (p = 0.50) or OCDS (p = 0.69) scores in the baseline moment, between patients who dropped out.

**Figure 1:**
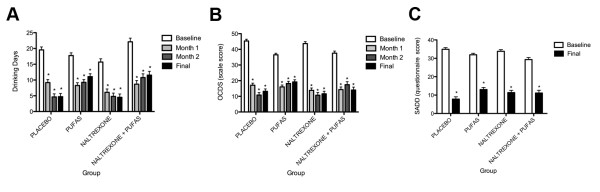
**Repeated Measures ANOVA of "Drinking Days", OCDS scale and SADD questionnaire**. (A) This graph represents the repeated measures ANOVA comparing the mean values of "drinking days" from baseline over time between the studied groups. (B) This graph represents the repeated measures ANOVA comparing the mean values of OCDS scale from baseline over time between the studied groups. (C) This graph represents the repeated measures ANOVA comparing the mean values of SADD questionnaire from baseline over time between the studied groups. * p values ≤ 0.001.

The accumulated data suggest that OCDS scores increases in relationship to the severity of the alcohol dependence syndrome [30]. In our study it was observed a highly significant correlation between the two instruments (+ 0.94). The initial and final mean values obtained for drinking days, SADD and OCDS in each group are demonstrated in Figure 1.

**Figure 2:**
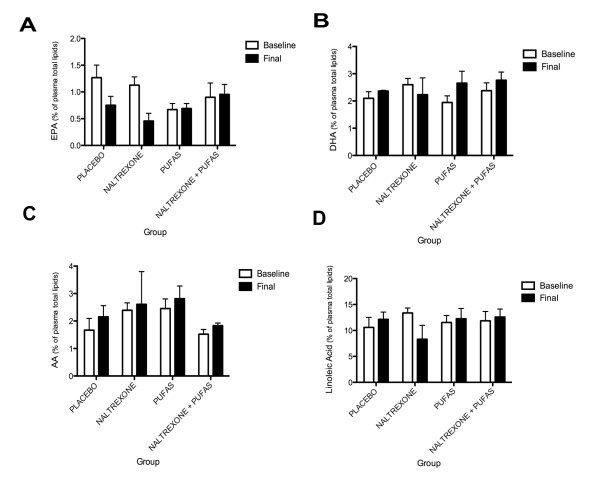
**Repeated Measures ANOVA of PUFAS serum measures**. (A) This graph represents the repeated measures ANOVA comparing the mean values of the percentage of EPA obtained from plasma total lipids, from baseline over time between the studied groups. (B) This graph represents the repeated measures ANOVA comparing the mean values of the percentage of DHA obtained from plasma total lipids, from baseline over time between the studied groups. (C) This graph represents the repeated measures ANOVA comparing the mean values of the percentage of AA obtained from plasma total lipids, from baseline over time between the studied groups. (D) This graph represents the repeated measures ANOVA comparing the mean values of the percentage of Linoleic Acid obtained from plasma total lipids, from baseline over time between the studied groups.

Regarding the laboratory work-up, there was no difference between the groups or over time for total cholesterol, HDL, LDL, alkaline phosphate, SGOT, SGPT, GGT and all other general exams performed. As for PUFAS dosage, the repeated measures ANOVA did not show a significant difference between groups and from the baseline to final visit dosages for EPA, DHA, AA or Linoleic acid. Figure 2 illustrates the results in each group.

No patients had any serious adverse event. Some of them (16, 21%, n = 80) complained of minor gastrointestinal disturbance.

## Discussion

In this study, the daily administration of PUFAS and Naltrexone, for a period of 3 months did not significantly differ from placebo in reducing the amount of alcohol ingestion, or OCDS and SADD scores in a group of severe alcohol dependent patient.

Overall all the four groups reduced significantly the amount of alcohol ingestion, dependence severity and compulsion for alcohol, when compared the baseline and the last visit values. This could be possibly explained by the severity of patient's dependence in the present trial. As patients had extremely severe dependence, high scores on SADD scale (mean SADD 33.55; ± 10.09), they might have benefited from the simple fact of been followed for a three-month period, received medical consultation, as well as "pharmacological treatment" (studied drugs or placebo). This could be an explanation of why the placebo group had similar improvement as the other treatment groups.

Several clinical studies have investigated the time and dose-dependent incorporation and successive washout of n-3 PUFAS in different biological tissues following dietary supplementation [31]. The evidence to date indicates that modest amounts of n-3 PUFAS can rapidly change the composition of fatty acids in blood cell membranes. A low-dose approach (1 g/day) appears justified by the concordance of biological and clinical findings. The International Society for the Study of Fatty Acids and Lipids (ISSFAL) for cardiovascular health, recommends a daily intake of EPA and DHA of 500 mg. Hibbeln et al. have proposed that the amount which would protect against major psychiatric diseases should be as high as 3.5 g per day [32]. The present trial supplemented 400 mg of EPA+DHA and 120 mg of GLA, which could not be an optimal dose for patients that are already depleted from those substances, as alcohol dependents. Therefore, it is possible that the amount of PUFAS administrated was not high enough to benefit the studied population, what could justify the lack of an expected positive effect.

**Figure 3:**
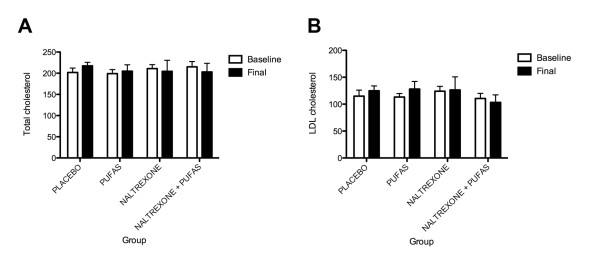
**Repeated Measures ANOVA of LDL and total cholesterol**. (A) This graph represents the repeated measures ANOVA comparing the mean values of Total cholesterol from baseline over time between the studied groups. (B) This graph represents the repeated measures ANOVA comparing the mean values of LDL cholesterol from baseline over time between the studied groups.

It is important to remark that even though no statistical differences were found between the groups, all PUFAS measurements increased in the groups to whom it was supplemented (Figure 2). Apparently, this increase was less intense in the group that also received naltrexone. Also, in the group that was given naltrexone alone, EPA, DHA and Linoleic acid dosages decreased. This effect could be related to the hipolipaemic effect cased by naltrexone, previously reported in both animal and human studies. Naltrexone prevented the stress-induced increase in total and LDL cholesterol in rats during stress caused by immobilization and a decrease in HDL-C levels [33]. Also, in patients treated with naltrexone, there was a significant decrease in total cholesterol (TC) concentration after 16 weeks of pharmacotherapy. Patients treated with naltrexone had lower mean TC (*P *< 0.03) and LDL-C (*P *< 0.01) than patients of the remaining pharmacotherapy groups [33]. In our study, even with only 12 weeks of treatment, a trend in naltrexone effects over PUFAS, TC and LDL-C could be observed (Figures 2 and 3).

It would have been interesting to obtain a dietary daily report of PUFAS ingestion by those patients. As they were treated on an outpatient basis and many kept drinking during the study, this could have helped to differentiate an inadequate diet from the deleterious effect of alcohol, in incorporating PUFAS to the cell membrane [34].

Another point to consider is the interaction between n-3 and n-6 series. Most of the studied conducted until now, administered only one PUFAS series, usually n-3. The patients enrolled in our study could be extremely depleted in the n-3 series, even more than from n-6 series. If that was true, they could benefit more from n-3 administration as monotherapy. It would be interesting, in future trials, to assess which would be a reference mean rate of PUFAS serum dosages in alcohol dependent patients, what would help to define which PUFAS series and dosage of supplementation might bring better outcomes.

An important consideration to be made is that, even though there was an increase on PUFAS serum dosages on the supplemented groups, the lack of adherence to the medications administered is still a concern. Despite being given calendar with dates and times (to check individually) and guided of its correct usage, some patients reported having completed the calendar at the day of consultation, because they forgot to check it in advance.

Comparisons also might have failed to reach statistical significance because the relatively small number of patients failed to lend enough power to the statistical analyses. As in other clinical trials for substance dependence, specially in an outpatient basis, most of the patients who start treatment do not accomplish to complete it [35]. Subsequent studies with more participants are needed to assess the possible benefit of the administration of polyunsaturated fatty acids to alcohol-dependent patients, either as monotherapy or in combination with naltrexone.

Our interpretation of the data should be taken in the context of our study's possible limitations. It should be pointed out that, to date, there is no data on the efficacy of PUFAS in alcohol dependence treatment. There are some studies aiming substance abuse population, but most of them have patient in use of more than one substance or medication such as methadone and antidepressants [6,36,37]. We aimed a very specific population of severe dependent patient that had no clinical comorbidities and in no use of other drugs of abuse as cocaine, heroin, cannabis or even benzodiazepines and other prescribed psychotropic medications.

## Study Limitations

Studies aiming substance abuse or dependence and PUFAS supplementation are very limited and to our knowledge, there is no combination proven to have positive effects on alcohol consumption or alcohol dependence severity.

In this study patient were given, 160 mg of EPA, 240 mg of DHA and 120 mg of GLA. The administration of both omega-3 and omega-6 aimed an increase of polyunsaturated and consequent decrease of saturated fatty acids membrane content. A low dosage was chosen because previous studies had demonstrated positive effects in other population. Another major concern in an outpatient trial is compliance, as patient would have to take a greater number of capsules in order to enlarge PUFAS supplementation.

EPA, DHA and GLA are major PUFAS components of cell membrane and have shown several benefits to human health. EPA and DHA are the two types of omega-3 fatty acids that serve as important precursors for lipid-derived modulators of cell signaling, gene expression and inflammatory processes [38]. GLA, a n-6 fatty acid have gained importance in the last four decades for its anti-inflammatory and anti-cancer actions. GLA and its metabolites also affect expression of various genes and play a significant role in immune functions and apoptosis [39]. Omega-6 fatty acids possibly also play a role as second messengers in the process of signal transduction across cell membranes [40].

Several clinical trials have supplemented PUFAS to the most diverse population. There is a wide range of dosage administered and combination of fatty acids. Some examples are a 6-month trial supplementing 80 mg EPA and 120 mg DHA that significantly protected an elderly population from a rise in serum triglycerides [41]; 80 mg AA daily for 3 weeks which increased the composition of AA, but did not decrease the composition of n-3 in young woman [42]; 324 mg EPA + 216 mg DHA + 480 mg LA and 258 mg GLA daily improved pulmonary status, inflammatory and anthropometric parameters in adults with cystic fibrosis [43]; and supplementation with only 200 mg DHA for 2 weeks induced an antioxidant effect, not achieved with 1600 mg [44].

There was an important drop-out from the start of the study until its completion 3 months later (from 80 patients to 43). A drop-out rate was expected, as the study was designed in an outpatient basis, but this loss overcame our expectations. Perhaps if they had additional visits, or associated psychotherapy, we could have had better results. As in majority of trials, patient who do not complete, tend to be the most severe ones. However, there was no statistical significant difference in the drop-out rate between the studied groups (PUFAS, Placebo, Naltrexone and Naltrexone + PUFAS) as previously discussed.

Another curious question is why there were no significant differences from baseline to final visit in EPA, DHA, AA and LA. It is possible that the given dosages were small and as the difference in PUFAS blood dosage was not statistical significant. We also have to consider that patient might not have taken their medication properly. They filled a calendar, as well as returned their empty medication container, but this does not necessary mean that they took the capsules as recommended. It is also important to take in account a possible hypolipaemic effect of Naltrexone as previously mentioned in the text.

## Conclusions

The administration of 120 mg of GLA and 400 mg EPA + DHA (160 mg EPA and 240 mg DHA), for 3 months did not significantly differ from placebo in reducing the amount of alcohol ingestion, or OCDS and SADD scores in a group of alcohol dependent patient. Subsequent studies with more participants are needed to assess the possible benefit of the administration of polyunsaturated fatty acids to alcohol-dependent patients, either as monotherapy or in combination with naltrexone.

## List of abbreviations

AA: Arachidonic acid; ALA_: _Alpha-linolenic acid; CNS: Central Nervous System; DHA: Docosahexaenoic acid; DPA: Docosapentaenoic acid; DSM-IV: Diagnostic and Statistical Manual of Mental Disorders; EPA: Eicosapentaenoic acid; GGT: Gamma-glutamyl transpeptidase; GLA: Gamma linolenic acid; HDL: High Density Lipoprotein; HIV: Human immunodeficiency virus; LA: Linoleic acid; LDL: Low Density Lipoprotein; n-3: Omega-3; n-6: Omega-6; OCDS: Obsessive compulsive drinking scale; PUFAS: Polyunsaturated fatty acids; SADD: Short Alcohol dependence data questionnaire; SGOT: Serum glutamic-oxaloacetic transaminase; SGPT: Serum glutamic-pyruvic transaminase; T3: Triiodothyronine; T4: Tetraiodothyronine or thyroxine; THC: Thetrahydrocannabinol; TSH: Thyroid-stimulating hormone; UNIFESP: Universidade Federal de São Paulo; VDRL: Venereal Disease Research Laboratory.

## Competing interests

The authors declare that they have no competing interests.

## Authors' contributions

The study was designed by RFSG and JCFG and JCFG wrote the protocol. Authors MNF and JCFG performed the clinical appointments and JKE was responsible for PUFAS quantification. The statistical analysis was made by MNF and RFSG, and MNF wrote the first draft of the manuscript. All authors contributed to and have approved the final manuscript.

## Pre-publication history

The pre-publication history for this paper can be accessed here:

http://www.biomedcentral.com/1472-6904/11/10/prepub
